# Femoral prosthesis fracture after hip arthroplasty revision: A Case Report and Review of Literature

**DOI:** 10.1097/MD.0000000000029811

**Published:** 2022-06-30

**Authors:** Long Yuan, Sen Li, Wanxiang Li, Jichao Bian, Yahui Bao, Xiaopeng Zhou, Yuanmin Zhang, Wang Li, Guodong Wang

**Affiliations:** a Department of Clinical Medicine, Jining Medical University, Jining, Shandong Province, China; b The Affiliated Hospital of Weifang Medical University, Weifang, Shandong Province, China; c Jining Second People’s Hospital, Jining, Shandong Province, China; d Department of Orthopedics, The Affiliated Hospital of Jining Medical University, Jining, Shandong Province, China.

**Keywords:** allogeneic bone plate, case report, prosthesis fracture, revision of hip joint, solution, Type B2 prosthesis loosening

## Abstract

A solution revision prosthesis has a multilayer microporous Porocoat coating, and the availability of multiple stem body sizes ensures that the prosthesis is adapted to each patient’s anatomical structure so that there a firm attachment with the bone cortex in the middle of the femur. Therefore, the Solution prosthesis is one of the most commonly used and most effective prostheses in total hip arthroplasty worldwide.

**Patient concerns:**

We reported a case of a 54-year-old female patient with periprosthetic femoral fractures after hip arthroplasty.

**Diagnosis:**

The case was identified as type B2 prosthesis loosening according to the Vancouver classification.

**Interventions:**

We performed revision surgery on her using the Solution prosthesis. Seven months after the surgery, the patient developed a mid-femoral prosthesis fracture for no apparent reason. We performed a second revision surgery of the hip joint and allogeneic bone plate fixation.

**Outcomes:**

The patient was satisfied with the treatment.

**Lessons:**

For patients with type B2 prosthesis loosening and prosthesis fracture, hip arthroplasty revision and an allogeneic bone plate could be used to ensure more stable support.

## 1. Introduction

The application of total hip arthroplasty (THA) is increasingly widespread. The number of primary THAs in the United States is expected to increase from approximately 253,000 cases in 2010 to approximately 572,000 cases in 2030.^[1]^ Currently, about 18% of THAs performed in the United States are revision surgeries, which means that there will be 5000 revision surgeries every year.^[2]^

Fracture of a femoral prosthesis in THA is a rare but catastrophic complication. Relevant studies have reported cases of prosthetic neck fracture after THAs.^[3–5]^ However, there have been few reports on body fractures of prostheses, especially after revision with Solution prostheses.

## 2. Case report

### 2.1. Chief complaints

A 54-year-old Chinese woman with pain in the right lower limb was sent to the Affiliated Hospital of Jining Medical University for further treatment.

### 2.2. History of present illness

The patient experienced pain in the right lower limb a week prior to the examination without any known cause, and the pain worsened in the 3 days leading up to her seeking medical treatment, with the inability to move out of bed, but she was not experiencing panic or chest tightness. She was treated with fluids at a local clinic; the exact name and dosage of the administered medication is not known; however, the pain did not ease. Therefore, she went to the hospital as an emergency patient. Radiography showed a fracture of the right upper femur and a broken artificial joint, and she was admitted to the hospital. Since the onset of the illness, the patient was in a good mental condition, with normal eating and sleeping, normal bowel function and urination, and no significant change in weight.

### 2.3. History of past illness

**Figure 1.**
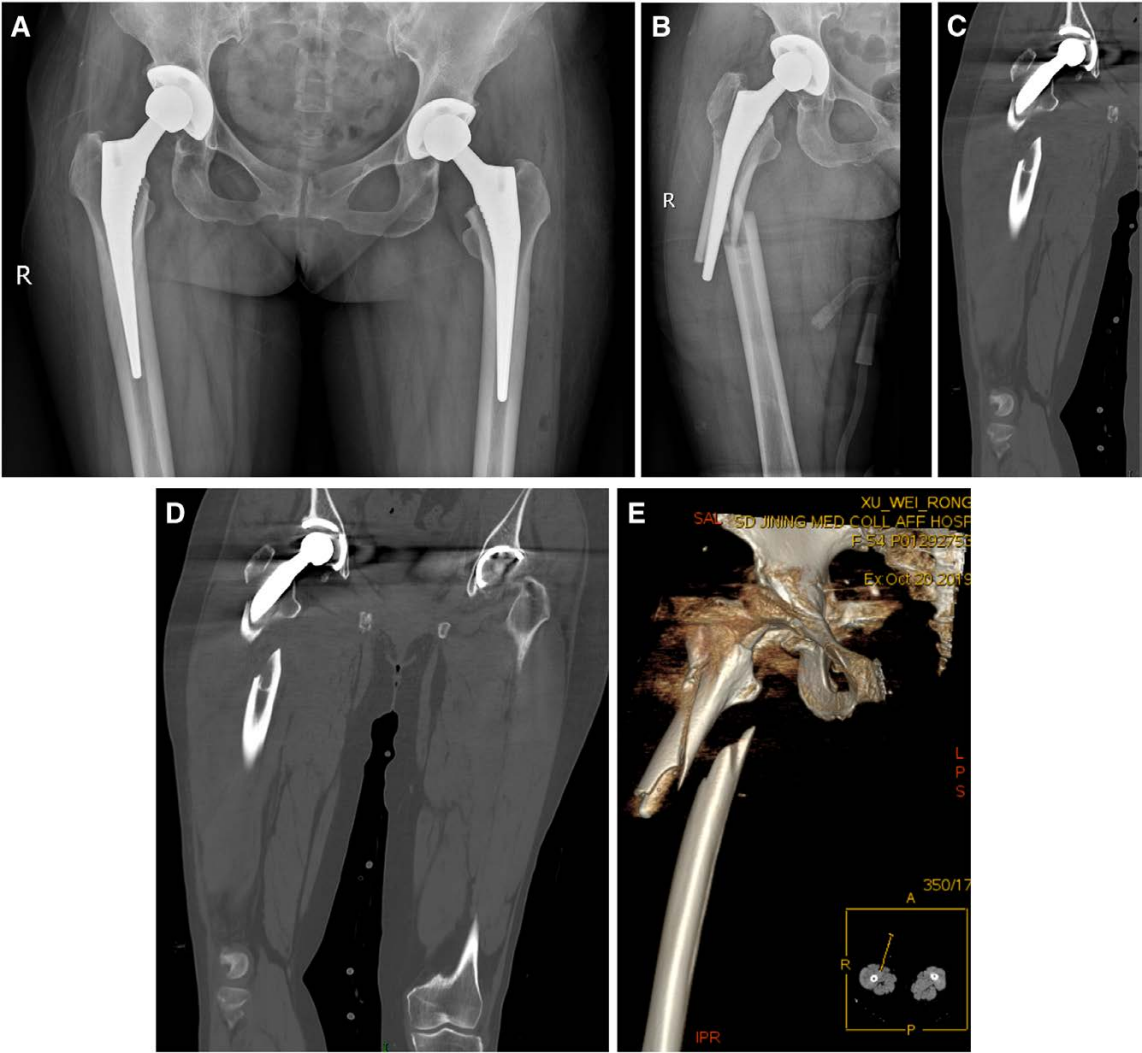
Imaging examination after bilateral THA and trauma. A: Radiography after bilateral THA; B: Radiography of the superior part of the right femur; C, D: Computed tomography (CT) scan of the right upper femur fracture after bilateral THA; E: 3 D CT reconstruction of the femoral fracture after right THA.

**Figure 2.**
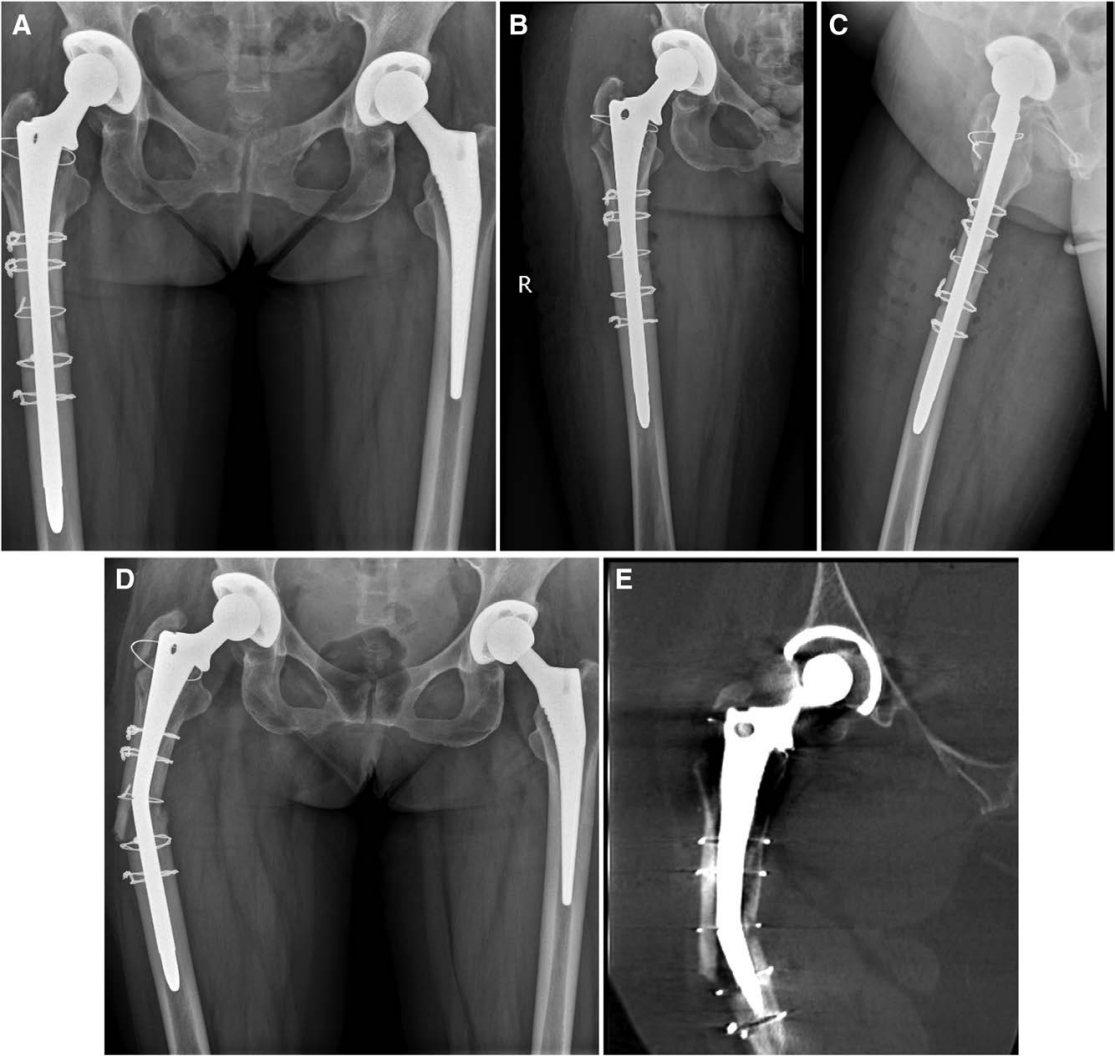
Radiographic analysis after the first revision of hip joint and entering the hospital this time. A–C: Orthostatic radiography of both hips after the revision of the right hip joint; anterior lateral radiography of the right hip; D, E: Orthostatic radiography and CT of the right upper femur fracture after the revision of the right hip joint.

The patient did not have a history of chronic diseases, such as hypertension, diabetes, or heart disease. Left hip arthroplasty was performed in our hospital more than 3 years ago, and right hip arthroplasty was done more than 2 years ago. She underwent revision of the hip joint prosthesis (right) and steel wire internal fixation of the femoral fracture in our hospital because of a car accident (Figs. 1, 2A–C). She has a history of blood transfusion, cephalosporin allergies, and mussel food allergies.

### 2.4. Personal and family history

The patient did not have a history of smoking, drinking, and exposure to toxic substances, dust, or radioactive substances. In addition, she did not have a family history of genetic or infectious diseases.

### 2.5. Physical examination

The patient’s temperature was 36.3 °C; her heart rate was 97 bpm; her respiratory rate was 20 breaths per minute; and her blood pressure was 145/107 mm Hg. A physical examination of the heart, lungs, and abdomen was negative. The right thigh was swollen and tender, and movement of the right lower limb was limited. The toes had good blood supply and moved normally.

### 2.6. Laboratory examinations

Other parameters such as routine analysis of blood and urine were normal.

### 2.7. Imaging examinations

Radiographic examination revealed a fracture of the right upper femur and a fracture of the artificial joint—changes after bilateral hip arthroplasty (Fig. 2D, E).

## 3. Final diagnosis

The final diagnoses of the case were right hip periprosthetic fracture, delayed healing of the right femoral fracture, right hip prosthesis fracture, and state after double hip replacement.

## 4. Treatment

**Figure 3.**
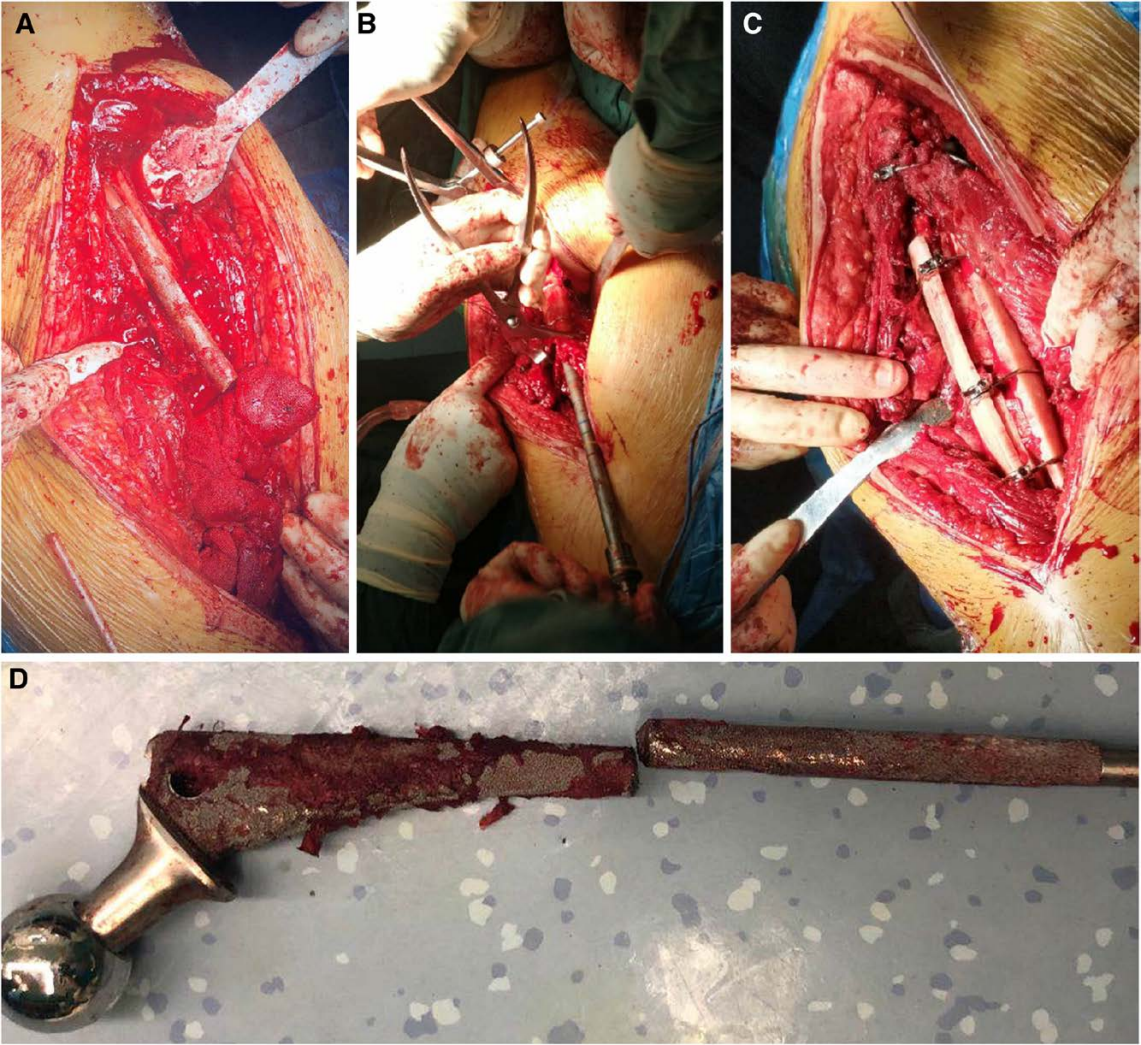
Pictures during surgery. A: The fracture ends at the femoral shaft are hardened, and the femoral prosthesis is broken at the fracture site; B: Temporary reduction and fixation of fracture, prebinding of steel wires, pulp cavity file and reaming; C: 2 allograft plates were placed on both sides of the broken end of the femur and fixed with 3 steel wires; D: A broken prosthesis was removed during surgery.

**Figure 4.**
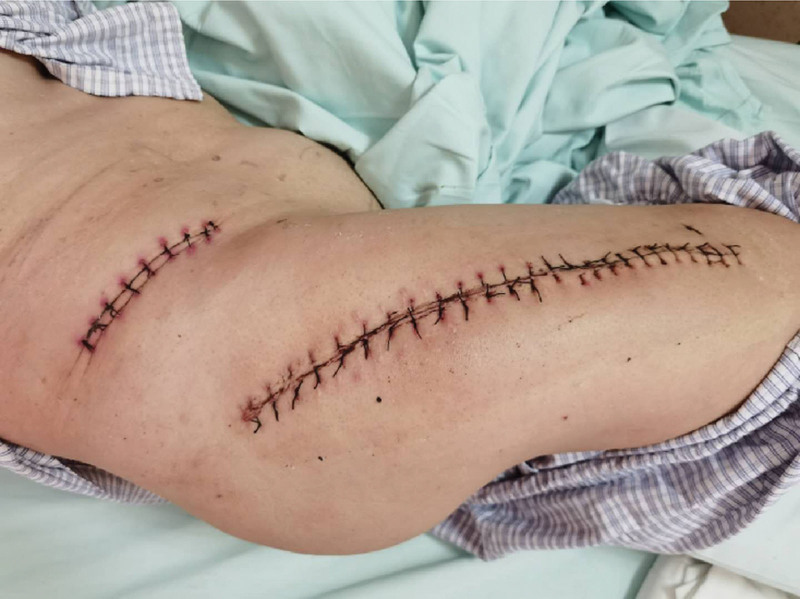
The incision healed well after the operation.

**Figure 5.**
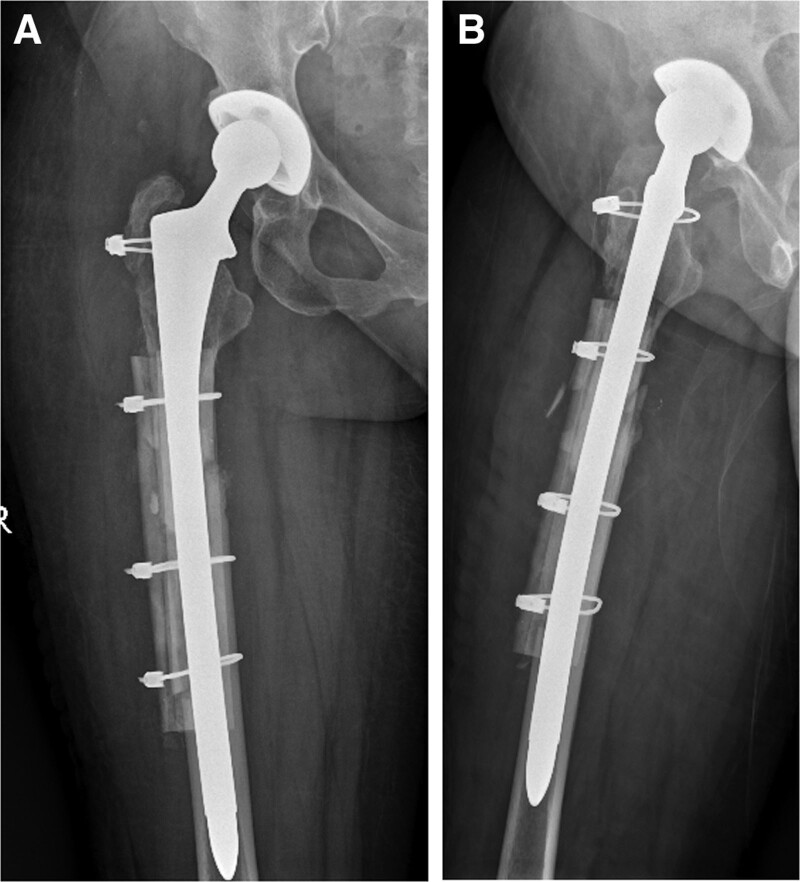
Radiographic analysis after the second revision of the right hip joint. A, B: Anterior and lateral radiographic positions after the second revision.

We decided to perform revision of the hip prosthesis and fix it with an allogeneic bone plate. During the operation, the acetabulum was fixed reliably without obvious wear; the fibrous connection at the greater trochanter of the femur had good continuity; the fractured end of the femoral shaft was hardened; and the femoral prosthesis was fractured at the fracture site. Therefore, it was necessary to open windows on both sides of the femur to remove the broken femoral stem prosthesis. The bone on the surface of the femoral stem grew well. First, the fracture was temporarily reduced and fixed with a steel wire, and then the medullary cavity was reamed with a medullary cavity file. Due to the narrow medullary cavity of the patient’s femur, we decided it was better to use a Solution handle medullary cavity file to enlarge the medullary cavity than the matched handle medullary cavity file. Next, the contents of the cavity were washed clean; the 13.5-mm Solution bowed femoral stem was inserted; and then a +8-mm metal head was placed, which could be reset without dislocation. After that, 2 allogeneic bone plates were placed on both sides of the femur fracture end; they were fixed with 3 steel wires, and 1 was fixed at the greater trochanter and femoral calcar (Fig. 3). The iliac bone was cut into strips and placed at the broken end of the femur. Fluoroscopy examination showed that the prosthesis was placed in a good position. Finally, a drainage tube was placed for flushing, and the wound was sutured owing to the satisfactory fracture reduction results. During the operation, the patient was given 300 mL of autologous blood, 4 units of allogeneic red blood suspension, and 400 mL of plasma. The patient recovered well after the operation (Figs. 4 and 5).

## 5. Outcome and follow-up

**Figure 6.**
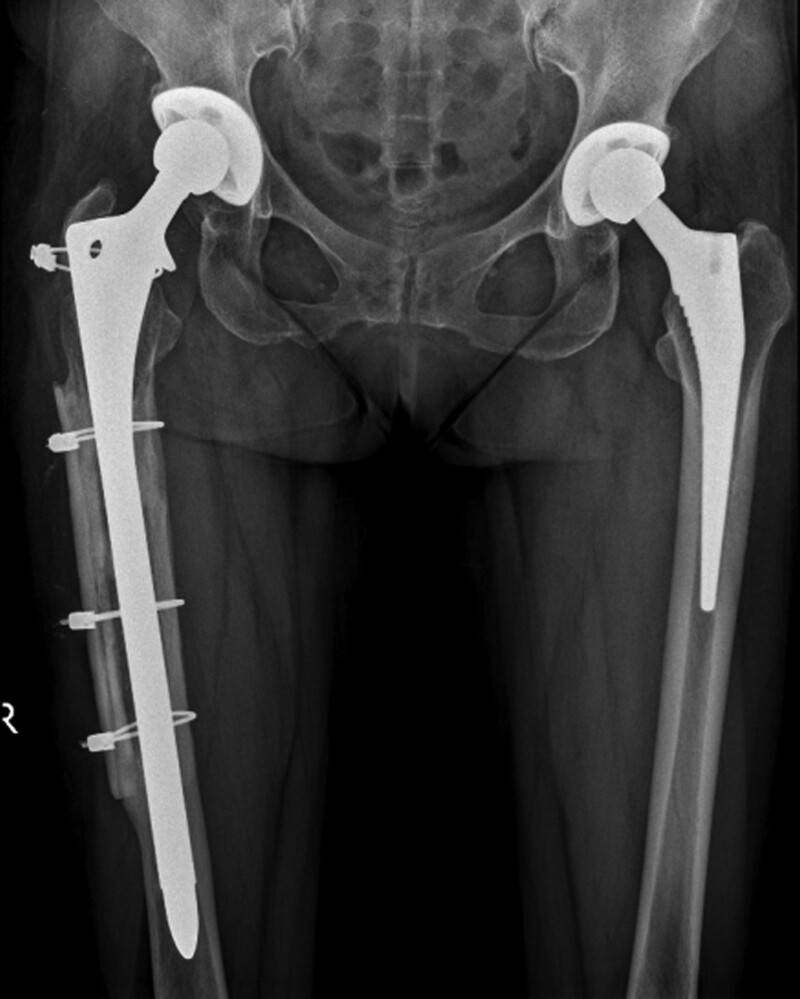
Radiographic analysis 1 year after the second revision of the right hip joint.

Reexamination was performed 1 year after the operation. Radiographic examination showed that the fracture healed well without obvious abnormalities (Fig. 6), and the hip joint activity returned to its normal level. The patient was satisfied with the results of the surgery.

## 6. Discussion

Modern THA is the most commonly used method for the treatment of end-stage hip osteoarthritis. Currently, the number of THAs is increasing, and the age of patients is decreasing year by year, which makes revision surgery increasingly common.^[6]^ Femoral prosthesis fracture is one of the most catastrophic complications after revision surgery, and the reason for prosthesis fracture has not yet been determined. Therefore, we considered the fracture as a stress prosthesis fracture in this case.

Atwood et al^[7]^ reported a case of Crowe type IV developmental dysplasia of the hip; the patient underwent THA and subtrochanteric osteotomy with nonunion at the osteotomy site and fracture of the distal prosthesis. It was considered an osteotomy nonunion, and the prosthesis was fatigued and fractured after long-term stress stimulation caused by femoral instability. Modular prostheses can achieve the optimal biomechanical effect by better restoring the anatomical structure of patients. However, modularity can also increase the amount of fretting, amount of corrosion, and the number of prosthesis fractures.^[8]^ Fretting of the connection interface induces fretting and crevice corrosion of the prosthesis, leading to microcracks in the corroded area and increasing the risk of dynamic fatigue fracture. Skendzel et al^[9]^ reported 2 cases of prosthetic neck fracture, and they emphasized that the use of a long varus neck in particular may have played a decisive role in the failure of such implants given that the bending moment of the long varus neck increased by more than 30% compared with the standard short neck. In our case, although the use of a long femoral stem after revision surgery would be more beneficial for femoral fracture healing, it could also increase the stress at the fracture site. The fractured femoral end was unstable after the first revision, and the prosthesis underwent a fatigue fracture because of the long-term stress stimulation caused by the instability of the femur. This case suggests the possibility of prosthesis body fracture after THA revision. Therefore, surgeons should pay attention to this issue because of the loss of bone mass in the medullary cavity after revision, and it is more difficult to carry out revision again.

A Solution prosthesis has a multilayer microhole wide coating, and the rough surface has a high friction coefficient, which is 33% higher than that of similar competitive products. It can form a firm attachment with the bone cortex in the middle of the femur and can obtain a “lock fit” of more than 4 to 6 cm to provide good stability.^[10, 11]^ The optimal pore size, pore gradient, and microparticle implantation can ensure bone ingrowth at the distal and proximal ends of the prosthesis and provide the best opportunity for rapid bone ingrowth.^[12]^ In this case, the bone loss in the epiphysis and femoral shaft was more severe in the second revision surgery, and the application of a Solution femoral stem was undoubtedly the most appropriate choice.

The use of an allogeneic bone plate can disperse the stress to the whole contact surface between the bone plate and the femur. Because allogeneic bone plate has an elastic modulus similar to that of the host bone, it can minimize the influence of stress shielding and stress concentration. Allogeneic bone plate can still have a good clinical effect when applied to a large number of bone defects. Lim et al^[13]^ conducted a prospective study with an average follow-up time of 5.4 ± 3.9 years. The results showed that 96% of patients achieved the connection between the allogeneic bone plate and the host femur. Park and moon ^[14]^ conducted medium- and long-term follow-up on the efficacy of allogeneic bone plate fixation of periprosthetic fractures. The average follow-up time was 8.6 years, and the final productivity was 94.7%, suggesting that an allogeneic bone plate can be used for the treatment of femoral defects and periprosthetic fractures. Previous studies have shown that the effect of allogeneic cortical strut transplantation was better than that of a metal plate. ^[15, 16]^

## 7. Conclusion

For patients with type B2 prosthesis loosening and prosthesis fracture, revision of the hip joint combined with allogeneic bone plate fixation can achieve a good therapeutic effect.

## Core Tip

Following hip revision, a female patient had a periprosthetic fracture and prosthesis fracture due to an unknown reason. We performed a second revision surgery to remove the fractured prosthesis. We placed a 13.5-mm Solution Bowed Stem with a +8-mm metal head and then 2 allograft plates on both sides of the broken femur. We secured them with 3 steel wires and then took the iliac bone and placed it in strips on the broken end of the femur. The patient was followed up and had recovered well. We believe that the treatment of this case is of great significance to patients with similar prosthesis fractures.

## Author contributions

Yuan L and Li S collected the data, imaging and operation reports and wrote the initial draft of the manuscript and subsequent revisions; Zhou XP and Wang L were the primary physicians during the patients’ inpatient stays; Li WX, Bao YH and Bian JC were involved in editing and overseeing the text; Zhang YM made the operation plan; Wang CQ were the chief surgeons of the patient; Wang GD was the senior author, and was responsible for oversight of the report and editing the manuscript; and all authors read and approved the final manuscript.
